# Frequency and Prognostic Value of KRAS, NRAS, and BRAF Mutations in Metastatic Colorectal Cancer: An Iranian Cohort Study

**DOI:** 10.34172/mejdd.3400

**Published:** 2026-04-30

**Authors:** Abbas Eshraghi, Mohammad Sadegh Ghasemi, Jamshid Vafaeimanesh, Mohammad Sina Mirjani, Mostafa Vahedian, Mohammad Reza Ahmadi

**Affiliations:** ^1^Department of Internal Medicine, Hematology & Oncology, School of Medicine, Qom University of Medical Sciences, Qom, Iran; ^2^Clinical Research of Development Center, Shahid Beheshti Hospital, Qom University of Medical Sciences, Qom, Iran; ^3^Department of Internal Medicine, School of Medicine, Qom University of Medical Sciences, Qom, Iran; ^4^Student Research Committee, Faculty of Medicine, Qom University of Medical Sciences, Qom, Iran; ^5^Research Center for Environmental Pollutants, Qom University of Medical Sciences, Qom, Iran

**Keywords:** Metastatic colorectal cancer, KRAS mutation, NRAS mutation, BRAF mutation

## Abstract

**Introduction:**

Colorectal cancer (CRC) is characterized by genetic mutations, particularly in the Rat sarcoma (RAS) and V-Raf Murine Sarcoma Viral Oncogene Homolog B (BRAF) oncogenes, which affect the prognosis and response to treatment. This study investigated the rates of Kristen Rat Sarcoma Viral oncogene homolog (K-RAS), neuroblastoma RAS viral oncogene homolog (N-RAS), and BRAF mutations and their association with 1-year survival among patients with metastatic CRC.

**Methods:**

This retrospective study included 83 patients with metastatic CRC from the oncology clinic at Shahid Beheshti Hospital, Qom, Iran. Demographic and clinical data, including mutation status, were collected. One-year patient survival was assessed.

**Results:**

In total, mutation frequencies were 47% for K-RAS, 14.5% for N-RAS, and 6% for BRAF. *BRAF* mutations were significantly more frequent in patients under 50 years of age, while *KRAS* and *NRAS* mutations showed no significant associations with age, sex, tumor site, or metastatic pattern. No significant association was observed between 1-year survival and KRAS, NRAS, or BRAF mutation status.

**Conclusion:**

BRAF mutations were significantly associated with an age of diagnosis under 50 years; however, neither BRAF, KRAS, nor NRAS mutations demonstrated a significant association with other clinicopathological characteristics or 1-year survival in this cohort.

## Introduction

 Colorectal cancer (CRC) is the third most prevalent malignancy worldwide, with an annual increase of 10.2% in the number of new cases.^1^ Despite advancements in screening programs, approximately 20-25% of patients with CRC still present with metastatic disease at the time of diagnosis.^2^ The 5-year survival rate for patients with non-metastatic CRC is estimated to be around 91%, whereas it dramatically declines to approximately 15% for those diagnosed with metastatic disease.^3^ Despite the significant progress in managing metastatic colorectal cancer (mCRC), recent years have shown no improvement in the median overall survival of these patients.^4^

 These findings underscore the need to elucidate the molecular and genetic profiles of mCRC to optimize therapeutic strategies.^5^ In CRC, the roles of mutated genes in the production of cancerous cells and in tumorigenesis are of significant importance. Mutations involving proto-oncogenes and their subsequent transformation into oncogenes are among the most critical pathophysiological alterations in these malignancies.^6^ Among these proto-oncogenes, mutations in the rat sarcoma (RAS) proto-oncogenes, including Kirsten rat sarcoma viral oncogene homolog (KRAS) and neuroblastoma rat sarcoma viral oncogene homolog (NRAS), are among the most often observed mutations in different malignancies, including colon adenocarcinoma, pancreatic cancer, lung cancer, and hematologic malignancies.^7^ Furthermore, the V-Raf Murine Sarcoma Viral Oncogene Homolog B (BRAF) mutation is associated with a poor prognosis and occurs in approximately 5% to 11% of patients with mCRC.^8^

 Several international studies have examined the clinical impact and frequency of these proto-oncogenes. However, there is limited evidence on this proto-oncogene in the Middle Eastern population, particularly in Iran.^9^ In this study, we examined the association between patient prognosis and mortality in patients with mCRC, as the identification of mutations in the NRAS, KRAS, and BRAF oncogenes is crucial for determining treatment response and prognosis.^10,11^ The purpose of this study was to determine the frequency of genetic variants in the K-RAS, N-RAS, and BRAF oncogenes, and the 1-year survival rate for each oncogene, in patients with mCRC.

## Materials and Methods

###  Study Design

 This retrospective cohort study included 83 patients diagnosed with mCRC at the oncology clinic of Shahid Beheshti Hospital, Qom, between 2019 and 2023. The diagnosis was confirmed by histological biopsy and imaging. Eligible patients underwent molecular testing for KRAS, NRAS, and BRAF mutations. Patients were monitored for 1 year following diagnosis to assess disease progression and survival through clinical visits and telephone follow-ups.

 Medical records provided patient demographic and clinical data, including age, sex, tumor site, histological features, and metastasis status. The inclusion criteria were a diagnosis of mCRC, referral to the oncology clinic at Shahid Beheshti Hospital in Qom between 2019 and 2023, and a history of chemotherapy. Researchers excluded patients who did not provide consent to participate, did not complete follow-up, or had incomplete data available.

###  Mutation Assessment

 After patient selection, tumor samples were analyzed as either fresh-frozen tissues or formalin-fixed, paraffin-embedded (FFPE) blocks containing at least 50% tumor cells. 4–5 sections with a thickness of 2–5 μm were prepared, and DNA was extracted using the QIAamp DNA FFPE Tissue Kit (Qiagen). For FFPE-derived DNA, a pre-amplification step was performed before high-resolution melting (HRM) analysis.

 Polymerase chain reaction (PCR) amplification was carried out under the following conditions: an initial denaturation at 95 °C for 11 minutes, followed by 30 cycles of denaturation at 95 °C for 30 seconds, annealing at 55 °C for 30 seconds, and extension at 72 °C for 30 seconds, with a final extension at 72 °C for 5 minutes. Successful amplification was confirmed by electrophoresis, which showed a 120 bp fragment for KRAS and NRAS (primers K12&13F and 13mt-R) and a 147 bp fragment for BRAF exon 15.

 The amplified fragments were analyzed using Gene Runner and MapViewer software to confirm the expected fragment length and restriction enzyme site (BglI) for restriction fragment length polymorphism (RFLP) analysis. Mutation status (wild-type or mutant) was determined by high-resolution melting (HRM) analysis. Samples with mutant profiles were further confirmed using allele-specific PCR, pyrosequencing, or direct DNA sequencing for BRAF V600E, KRAS, and NRAS mutations.

###  Statistical Analysis

 Independent t-tests or ANOVA were performed for continuous variables to assess the association between mutation status and clinicopathological parameters. Conversely, categorical variables were analyzed using Chi-square or Fisher’s exact tests as appropriate. Binary logistic regression analysis was used to identify independent predictors of mutation status. All statistical analyses were conducted in RStudio, and *P* < 0.05 was considered statistically significant.

###  Ethical Approval

 This study was approved by the Research Ethics Committee of Qom University of Medical Sciences (Approval ID: IR.MUQ.REC.1401.207, Approval Date: January 8, 2023).

## Results

 During the research period, 83 patients met the inclusion criteria. The mean age of the patients was 54.87 ± 12.78 years. The patients’ ages ranged from 23 to 85 years. A total of 30 female patients (35.4%) and 53 male patients (64.6%) were studied. The results indicated that mutations in the K-RAS gene (39 patients, 47%) were the most frequent among the oncogenes examined. Mutations in the N-RAS gene (12 patients, 14.5%) and BRAF gene (5 patients, 6%) were observed at lower frequencies.

 Among patients with gene mutations, the lowest mean age was associated with BRAF mutations (40.2 years), and the highest with K-RAS mutations (57.59 years). A significant difference in mean age was observed between patients with mutated and wild-type BRAF genes (*P* = 0.038) (Table 1). However, findings regarding BRAF mutations should be interpreted with caution given the very small number of BRAF-mutant patients (n = 5).

**Table 1:** Baseline clinicopathological characteristics and one-year survival outcome

**Characteristics**		**KRAS**			**NRAS**			**BRAF**		
		**Mutant (n=39)**	**Wild (n=44)**	***P***	**Mutant (n=12)**	**Wild (n=71)**	***P***	**Mutant (n=5)**	**Wild (n=78)**	***P***
Primary tumor location	Left	29 (74.4%)	31 (70.5%)	0.079*	9 (75%)	51 (71.8%)	0.204*	3 (60%)	57 (73.1%)	0.366*
	Right	5 (12.8%)	12 (27.3%)		1 (8.3%)	16 (22.5%)		1 (20%)	16 (20.5%)	
	Unspecified	5 (12.8%)	1 (2.3%)		2 (16.7%)	4 (5.6%)		1 (20%)	5 (6.4%)	
Liver metastasis	No	6 (15.4%)	6 (13.6%)	0.821#	2 (16.7%)	10 (14.1%)	0.683*	1 (20%)	11 (14.1%)	0.552*
	Yes	33 (84.6%)	38 (86.4%)		10 (83.3%)	61 (85.9%)		4 (80%)	67 (85.9%)	
Lung metastasis	No	25 (64.1%)	36 (81.8%)	0.068#	8 (66.7%)	53 (74.6%)	0.724*	4 (80%)	57 (73.1%)	1*
	Yes	14 (35.9%)	8 (18.2%)		4 (33.3%)	18 (25.4%)		1 (20%)	21 (26.9%)	
Bone metastasis	No	38 (97.4%)	42 (95.5%)	1*	12 (100%)	68 (95.8%)	1*	5 (100%)	75 (96.2%)	1*
	Yes	1 (2.6%)	2 (4.5%)		0 (0%)	3 (4.2%)		0 (0%)	3 (3.8%)	
Lymph nodes metastasis	No	36 (92.3%)	41 (93.2%)	1*	11 (91.7%)	66 (93%)	1*	4 (80%)	73 (93.6%)	0.319*
	Yes	3 (7.7%)	3 (6.8%)		1 (8.3%)	5 (7%)		1 (20%)	5 (6.4%)	
Peritoneal metastasis	No	37 (94.9%)	36 (81.8%)	0.094*	10 (83.3%)	63 (88.7%)	0.633*	5 (100%)	68 (87.2%)	1*
	Yes	2 (5.1%)	8 (18.2%)		2 (16.7%)	8 (11.3%)		0 (0%)	10 (12.8%)	
Age	< 50	9 (23.1%)	17 (38.6%)	0.127#	6 (50%)	20 (28.2%)	0.131#	4 (80%)	22 (28.2%)	0.032*
	≥ 50	30 (76.9%)	27 (61.4%)		6 (50%)	51 (71.8%)		1 (20%)	56 (71.8%)	
Sex	Male	22 (56.4%)	31 (70.5%)	0.184#	8 (66.7%)	45 (63.4%)	1*	2 (40%)	51 (65.4%)	0.346*
	Female	17 (43.6%)	13 (29.5%)		4 (33.3%)	26 (36.6%)		3 (60%)	27 (34.6%)	
One-year survival	Alive	23 (76.7%)	30 (68.2%)	0.520#	9 (75%)	44 (62%)	0.522*	2 (40%)	51 (65.4%)	0.346*
	Death	16 (23.3%)	14 (31.8%)		3 (25%)	27 (38.0%)		3 (60%)	27 (34.6%)	
Age (mean ± SD)		57.59 ± 12.49	52.45 ± 12.69	0.067**	51.7 ± 15.85	55.39 ± 12.25	0.461**	40.20 ± 11.71	55.81 ± 12.33	0.038**

* = Fisher # = Chi-square ** = t-test. Bold numbers indicate statistically significant p-values (*P* < 0.05).

 The distribution of KRAS, NRAS, and BRAF mutations did not differ significantly by sex. KRAS mutations were present in 43.6% of females and 56.4% of males (*P* = 0.184). NRAS mutations occurred in 33.3% of females and 66.7% of males (*P* = 1.000). BRAF mutations were detected in 60% of females and 40% of males (*P* = 0.346). Overall, no significant association was observed between sex and mutation status for any of the three genes (Table 1).

 The left colon was the predominant primary tumor site across KRAS (74.4%), NRAS (75%), and BRAF (60%) mutation groups, with no significant associations between primary tumor location and mutation status (KRAS: *P* = 0.079; NRAS: *P* = 0.204; BRAF: *P* = 0.366). The liver was the predominant site of metastasis across all mutation groups: KRAS (84.6%), NRAS (83.3%), and BRAF (80%). No significant association was found between mutation status and liver metastasis for KRAS (*P* = 0.821), NRAS (*P* = 0.683), or BRAF (*P* = 0.552) (Table 1).

 One-year survival was evaluated in patients according to KRAS, NRAS, and BRAF mutation status. Overall, there were no statistically significant associations between KRAS (*P* = 0.520), NRAS (*P* = 0.522), or BRAF (*P* = 0.346) mutation status and 1-year survival (Table 1).

 Among mutation-positive subgroups, none of the clinicopathological characteristics showed a significant association with 1-year survival (Table 2). Similarly, no clinicopathological factor was significantly associated with 1-year survival in wild-type gene subgroups (Table 3). In the overall population, only liver metastasis demonstrated a statistically significant association with 1-year survival (*P* = 0.048) (Table 4).

**Table 2:** Association between clinicopathological characteristics and 1-year survival in mutant gene subgroups

**Characteristics**		**KRAS-Mutant**			**NRAS-Mutant**			**BRAF-Mutant**		
		**Alive (n=23)**	**Death (n=16)**	***P***	**Alive (n=9)**	**Death (n=3)**	***P***	**Alive (n=2)**	**Death (n=3)**	***P***
Primary tumor location	Left	15 (65.2%)	14 (87.5%)	0.158*	7 (77.8%)	2 (66.7%)	0.618*	1 (50%)	2 (66.7%)	1*
	Right	5 (21.7%)	0 (0%)		1 (11.1%)	0 (0%)		1 (50%)	0 (0%)	
	Unspecified	3 (13%)	2 (12.5%)		1 (11.1%)	1 (33.3%)		0 (0%)	1 (33.3%)	
Liver metastasis	No	6 (26.1%)	0 (0%)	0.064*	2 (22.2%)	0 (0%)	1*	1 (50%)	0 (0%)	0.400*
	Yes	17 (73.9%)	16 (100%)		7 (77.8%)	3 (100%)		1 (50%)	3 (100%)	
Lung metastasis	No	15 (65.2%)	10 (62.5%)	0.862#	5 (55.6%)	3 (100%)	0.491*	2 (100%)	2 (66.7%)	1*
	Yes	8 (34.8%)	6 (37.5%)		4 (44.4%)	0 (0%)		0 (0%)	1 (33.3%)	
Bone metastasis	No	23 (100%)	15 (93.8%)	0.410*	9 (100%)	3 (100%)	1*	2 (100%)	3 (100%)	1*
	Yes	0 (0%)	1 (6.2%)		0 (0%)	0 (0%)		0 (0%)	0 (0%)	
Lymph nodes metastasis	No	20 (87%)	16 (100%)	0.255*	8 (88.9%)	3 (100%)	1*	1 (50%)	3 (100%)	0.400*
	Yes	3 (13%)	0 (0%)		1 (11.1%)	0 (0%)		1 (50%)	0 (0%)	
Peritoneal metastasis	No	21 (91.3%)	16 (100%)	0.503*	7 (77.8%)	3 (100%)	1*	2 (100%)	3 (100%)	1*
	Yes	2 (8.7%)	0 (0%)		2 (22.2%)	0 (0%)		0 (0%)	0 (0%)	
Age	< 50	6 (26.1%)	3 (18.8%)	0.711*	4 (44.4%)	2 (66.7%)	1*	1 (50%)	3 (100%)	0.400*
	≥ 50	17 (73.9%)	13 (81.2%)		5 (55.6%)	1 (33.3%)		1 (50%)	0 (0%)	
Sex	Female	13 (56.5%)	4 (25%)	0.099*	2 (22.2%)	2 (66.7%)	0.236*	1 (50%)	2 (66.7%)	1*
	Male	10 (43.5%)	12 (75%)		7 (77.8%)	1 (33.3%)		1 (50%)	1 (33.3%)	

* = Fisher # = Chi-square

**Table 3:** Association between clinicopathological characteristics and 1-year survival in wild-type gene subgroups

**Characteristics**		**KRAS-Wild**			**NRAS-Wild**			**BRAF-Wild**		
		**Alive (n=30)**	**Death (n=14)**	***P***	**Alive (n=44)**	**Death (n=27)**	***P***	**Alive (n=51)**	**Death (n=27)**	***P***
Primary tumor location	Left	20 (66.7%)	11 (78.6%)	0.809*	28 (63.6%)	23 (85.2%)	0.125*	34 (66.7%)	23 (85.2%)	0.222*
	Right	9 (30%)	3 (21.4%)		13 (29.5%)	3 (11.1%)		13 (25.5%)	3 (11.1%)	
	Unspecified	1 (3.3%)	0 (0%)		3 (6.8%)	1 (3.7%)		4 (7.8%)	1 (3.7%)	
Liver metastasis	No	5 (16.7%)	1 (7.1%)	0.647*	9 (20.5%)	1 (3.7%)	0.077*	10 (19.6%)	1 (3.7%)	0.086*
	Yes	25 (83.3%)	13 (92.9%)		35 (79.5%)	26 (96.3%)		41 (80.4%)	26 (96.3%)	
Lung metastasis	No	24 (80%)	12 (85.7%)	1*	34 (77.3%)	19 (70.4%)	0.516#	37 (72.5%)	20 (74.1%)	0.885#
	Yes	6 (20%)	2 (14.3%)		10 (22.7%)	8 (29.6%)		14 (27.5%)	7 (25.9%)	
Bone metastasis	No	28 (93.3%)	14 (100%)	1*	42 (95.5%)	26 (96.3%)	1*	49 (96.1%)	26 (96.3%)	1*
	Yes	2 (6.7%)	0 (0%)		2 (4.5%)	1 (3.7%)		2 (3.9%)	1 (3.7%)	
Lymph nodes metastasis	No	28 (93.3%)	13 (92.9%)	1*	40 (90.9%)	26 (96.3%)	0.643*	47 (92.2%)	26 (96.3%)	0.654*
	Yes	2 (6.7%)	1 (7.1%)		4 (9.1%)	1 (3.7%)		4 (7.8%)	1 (3.7%)	
Peritoneal metastasis	No	26 (86.7%)	10 (71.4%)	0.242*	40 (90.9%)	23 (85.2%)	0.468*	45 (88.2%)	23 (85.2%)	0.731*
	Yes	4 (13.3%)	4 (28.6%)		4 (9.1%)	4 (14.8%)		6 (11.8%)	4 (14.8%)	
Age	< 50	11 (36.7%)	6 (42.9%)	0.694#	13 (29.5%)	7 (25.9%)	0.742#	16 (31.4%)	6 (22.2%)	0.393#
	≥ 50	19 (63.3%)	8 (57.1%)		31 (70.5%)	20 (74.1%)		35 (68.6%)	21 (77.8%)	
Sex	Female	8 (26.7%)	5 (35.7%)	0.540#	19 (43.2%)	7 (25.9%)	0.143#	20 (39.2%)	7 (25.9%)	0.240#
	Male	22 (73.3%)	9 (64.3%)		25 (56.8%)	20 (74.1%)		31 (60.8%)	20 (74.1%)	

* = Fisher; # = Chi-square

**Table 4:** Clinicopathological factors associated with 1-year survival in all patients

**Characteristics**		**Alive (n=53)**	**Death (n=30)**	***P***
Primary tumor location	Left	35 (66%)	25 (83.3%)	0.209*
	Right	14 (26.4%)	3 (10%)	
	Unspecified	4 (7.6%)	2 (6.7%)	
Liver metastasis	No	11 (20.8%)	1(3.3%)	0.048*
	Yes	42 (79.2%)	29 (96.7%)	
Lung metastasis	No	39 (73.6%)	22 (73.3%)	0.980#
	Yes	14 (26.4%)	8 (26.7%)	
Bone metastasis	No	51 (96.2%)	29 (96.7%)	1*
	Yes	2 (3.8%)	1(3.3%)	
Lymph nodes metastasis	No	48 (90.6%)	29 (96.7%)	0.410*
	Yes	5 (9.4%)	1(3.3%)	
Peritoneal metastasis	No	47 (88.7%)	26 (86.7%)	1*
	Yes	6 (11.3%)	4 (13.3%)	
Age	< 50	17 (32.1%)	9 (30%)	0.845#
	≥ 50	36 (67.9%)	21 (70%)	
Sex	Female	21 (39.6%)	9 (30%)	0.381#
	Male	32 (60.4%)	21 (70%)	

* = Fisher; # = Chi-square Bold numbers indicate statistically significant p-values (*P* < 0.05).

Table 5 presents the results of the multivariate logistic regression analysis for factors associated with 1-year survival. None of the evaluated variables showed a statistically significant association with 1-year survival (all *P* > 0.05).

**Table 5:** Multivariate analysis of factors associated with 1-year survival

**Variables**		OR*	***P***
Sex	Female vs Male	1.39 (0.44-4.60)	0.579
KRAS	Mutant vs Wild	0.47 (0.14-1.45)	0.192
NRAS	Mutant vs Wild	2.59 (0.57-15.95)	0.250
BRAF	Mutant vs Wild	0.20 (0.01-2.22)	0.202
Primary tumor location	Right vs Left	2.90 (0.67-16.42)	0.179
Primary tumor location	Unspecified vs Left	0.71 (0.06-9.89)	0.780
Liver metastasis	Yes vs No	0.18 (0.01-1.41)	0.157
Lung metastasis	Yes vs No	1.21 (0.36-4.21)	0.757
Bone metastasis	Yes vs No	1.26 (0.07-44.43)	0.879
Lymph nodes metastasis	Yes vs No	1.80 (0.16-44.54)	0.658
Peritoneal metastases	Yes vs No	0.33 (0.05-1.98)	0.229
Age	≥ 50 vs < 50	0.72 (0.11-4.53)	0.727

* = Binary logistic regression

## Discussion

 The purpose of this study was to determine the frequency of KRAS, NRAS, and BRAF mutations in patients with mCRC and to evaluate their association with clinicopathological characteristics and 1-year survival. In this study, we found that KRAS mutations were the most frequently observed among the examined oncogenes, while NRAS and BRAF mutations were less common. Based on our analysis, the left-sided colon was the predominant primary tumor site across all mutation types, and the liver was the most frequent site of metastasis. We did not find significant associations between mutation status or clinicopathological features and 1-year survival, except for liver metastasis, which was linked to survival in the overall population.

 The relatively high prevalence of KRAS mutations in our cohort may have important therapeutic implications, as RAS-mutant tumors are resistant to anti-EGFR monoclonal antibodies.^12^ It may therefore require alternative targeted treatment strategies. Furthermore, evidence indicates that the mutational landscape of colorectal cancer varies across racial, ethnic, and geographic populations.^13,14^ This fact highlights the importance of region-based molecular epidemiology when interpreting mutation frequencies and developing precision oncology strategies.

 The mutation rates for the K-RAS, N-RAS, and BRAF genes were 47%, 14.5%, and 6%, respectively, according to this study. These rates are comparable to those reported in other studies, such as the 33% K-RAS mutation rate in the study by Trevisiol and colleagues in Italy and the 21.6% K-RAS mutation rate in the study by Mikami and co-workers in Japan.^15,16^ Variation across study populations and larger sample sizes in those studies may be the cause of these discrepancies. For instance, the mutation rates were 53% and 39.3% in studies conducted by Ryan et al. and Neumann et al, respectively.^17,18^ Furthermore, Ahmadi and coleagues conducted a study in Iran that revealed a K-RAS mutation rate of 52%, which is comparable to the frequency reported in the current study (47%).^19^ Similar research populations could help explain this resemblance.

 Similar to Dogan and others, most patients with KRAS mutation in our study had left-sided tumors; however, unlike their findings, we did not observe a statistically significant association between tumor location and 1-year survival.^20^ Regarding liver metastasis, both studies reported no significant association with survival in univariate or multivariate analyses, indicating a consistent pattern between studies. These similarities and differences may be attributed to variations in patient selection, as Dogan and others exclusively included KRAS-mutant metastatic cases. In contrast, our study comprised both KRAS-mutant and wild-type patients.

 The N-RAS mutation rate in Ahmadi et al.’s study was 4%, which is lower than the rate observed in the present study (14%). This difference may be caused by differences in laboratory techniques and detection methods, as variations in sequencing technologies can affect the sensitivity for identifying mutations.^19^ Additionally, in the study by Eshraghi and others, approximately 14.8% of the studied population had N-RAS mutations.^21^ The mutation rate in our study was 14.5%, which is comparable to that reported by Eshraghi and colleagues.

 Ros and co-workers found BRAF mutations in 12% of patients with metastatic colorectal cancer. They found that a BRAF mutation was associated with a worse prognosis.^22^ Although the frequency of this oncogene was lower (6%) in the current study’s general population, it was higher among deceased patients. However, this finding was not statistically significant, possibly because of differences in sample sizes between the two investigations. Although not statistically significant, the higher frequency of BRAF mutations among deceased patients may indicate a clinically relevant trend. BRAF-mutant colorectal cancer is characterized by aggressive tumor biology, increased metastatic potential, and inferior survival outcomes.^23,24^ Therefore, the absence of statistical significance in our study may reflect limited statistical power rather than the absence of a biological effect.

 A statistically significant difference in BRAF gene mutation was observed by age, with a higher percentage of patients under 50 years old exhibiting this mutation (*P* = 0.032). However, no substantial association with sex was identified. On the other hand, a study by D. Booker and colleagues found an association between the frequency of this mutation and sex, with a higher frequency in females.^25^ Differences in methodology across studies, such as variations in diagnostic techniques or criteria for identifying the BRAF mutation, may account for this discrepancy.

 In our study, the majority of BRAF-mutant patients were younger than 50 years, which aligns with findings from Soto et al, who reported a higher frequency of BRAF mutations among patients under 65 years.^26^ However, in the COALA study from Australia, a higher proportion of BRAF mutations was observed in older patients, suggesting that the age distribution of BRAF-mutant colorectal cancer may vary across populations.^27^

 An important finding of the present study was the association between liver metastasis and 1-year survival. The liver is the most common site of metastatic spread in CRC and remains a major determinant of prognosis because hepatic tumor burden directly affects treatment eligibility and overall disease progression. Our findings suggest that anatomical disease extent may exert a substantial influence on short-term survival, potentially exceeding that of individual molecular alterations in certain clinical settings.^28-30^

 This study revealed no significant association between mutations in the genes under investigation and 1-year patient survival. The findings of this study align with the research results obtained by Ahmadi et al. regarding the K-RAS and N-RAS genes.^19^ However, the study conducted by D. Booker et al. revealed an association between the frequency of BRAF mutations and reduced overall patient survival.^25^ The observed differences in findings may be explained by variations in sample size and a longer follow-up period. These findings suggest that while these specific genetic alterations may not strictly dictate short-term (1-year) survival, their implications for long-term prognosis require further longitudinal investigation.

## Limitations

 The retrospective design, single-center setting, and relatively small sample size, particularly for NRAS and BRAF mutations, reduce statistical power and may limit generalizability. The follow-up period was limited to 1 year. Some clinical data, including detailed treatment regimens and comorbidities, were incomplete. Larger studies with larger sample sizes and more extended follow-up periods in the Iranian population are needed to validate these findings.

 Despite these limitations, this study has potential implications for identifying prognostic factors, particularly genetic alterations that may influence overall survival. Detecting these mutations could improve clinical planning and the personalized management of patients with mCRC.

## Conclusion

 This study found a significant association between BRAF mutations and an age of less than 50 years in patients with mCRC. However, no statistically significant associations were observed between BRAF mutations and other demographic factors or 1-year survival. Similarly, no significant associations were found between K-RAS and N-RAS mutations and demographic factors or 1-year survival. Despite the study’s limitations, these findings contribute to the understanding of the molecular epidemiology of mCRC in the region and may support future development of personalized targeted therapies.
